# Lesser Grain Borers, *Rhyzopertha dominica*, Select Rough Rice Kernels with Cracked Hulls for Reproduction

**DOI:** 10.1673/031.012.3801

**Published:** 2012-03-23

**Authors:** Nickolas G. Kavallieratos, Christos G. Athanassiou, Frank H. Arthur, James E. Throne

**Affiliations:** ^1^ Laboratory of Agricultural Entomology, Department of Entomology and Agricultural Zoology, Benaki Phytopathological Institute; 8 Stefanou Delta str., 14561, Kifissia, Attica, Greece; ^2^ Laboratory of Entomology and Applied Zoology, Department of Agriculture, Crop Production and Rural Environment, University of Thessaly, Phytokou str., Nea lonia, 38446, Magnissia, Greece; ^3^ USDA-ARS Center for Grain and Animal Health Research, 1515 College Avenue, Manhattan, KS 66502–2736, USA

**Keywords:** selection, stored-product, varietal resistance

## Abstract

Tests were conducted to determine whether the lesser grain borer, *Rhyzopertha dominica* (F.) (Coleoptera: Bostrychidae), selects rough rice (*Oryza sativa* L. (Poales: Poaceae)) kernels with cracked hulls for reproduction when these kernels are mixed with intact kernels. Differing amounts of kernels with cracked hulls (0, 5, 10, and 20%) of the varieties Francis and Wells were mixed with intact kernels, and the number of adult progeny emerging from intact kernels and from kernels with cracked hulls was determined. The Wells variety had been previously classified as tolerant to *R. dominica*, while the Francis variety was classified as moderately susceptible. Few F 1 progeny were produced in Wells regardless of the percentage of kernels with cracked hulls, few of the kernels with cracked hulls had emergence holes, and little firass was produced from feeding damage. At 10 and 20% kernels with cracked hulls, the progeny production, number of emergence holes in kernels with cracked hulls, and the amount of firass was greater in Francis than in Wells. The proportion of progeny emerging from kernels with cracked hulls increased as the proportion of kernels with cracked hulls increased. The results indicate that *R. dominica* select kernels with cracked hulls for reproduction.

## Introduction

Kernel hardness in grains has been associated with tolerance or resistance to stored-product insects; in general, progeny production decreases as kernel hardness increases (Throne et al. 2000). However, kernel hardness is not a constant indicator of tolerance among different grains or different varieties of grains (McGaughey et al. 1990; Pomeranz et al. 1988). McGaughey et al. (1990) found that hardness can strongly influence the ability of *Sitophilus oryzae* to reproduce in stored wheat. However, Bhatia and Gupta (1969), Amos et al. (1986), Sinha et al. (1988), and Toews et al. (2000) showed that grain hardness was not related to reproduction by *Rhyzopertha dominica* (F.) (Coleoptera: Bostrychidae). The presence of cracked kernels often increases progeny production of external stored-grain beetle pests (e.g., Athanassiou et al. 2010) and internal pests such as the lesser grain borer, *R. dominica* (Throne et al. 2000).

*Rhyzopertha dominica* is one of the most important pests of stored rice *Oryza sativa* L. (Poales: Poaceae) worldwide. The female lays an egg outside the kernel, and the newly hatched larva bores into the kernel where it completes development. Harvested rice is enclosed in a hull formed by two leaves called the palea and the lemma, which protects the kernel. As with other grains, different varieties of rice vary in their susceptibility to stored-product insects (Russell 1968; Cogburn 1974). Chanbang et al. (2008a) found that varieties with more cracks and splits in the hull (e.g., variety Francis) provided a pathway for entry of neonate *R. dominica*, and eventual emergence of adults was greater in those varieties compared with varieties with fewer cracks and splits in the hull (e.g., variety Wells). Chanbang et al. (2008b) tested varietal resistance by placing an egg of *R. dominica* on single kernels of different varieties of rice. They did not test the ability of adult female *R. dominica* to seek out kernels with cracked hulls for oviposition or the ability of neonates to seek out kernels with cracked hulls for feeding and development, particularly when those kernels were mixed in a larger mass of sound kernels with intact hulls. We hypothesize that kernels with cracked hulls are more likely to be sought out by females for oviposition or to be sought out by neonates for feeding and development. So far, there are no published data that examine this hypothesis. Thus, in the present paper we determined emergence of progeny adults from kernels with cracked hulls that were mixed with intact kernels as an indicator of which kernels were selected for oviposition or feeding and development.

## Materials and Methods

Rice varieties Wells and Francis from the 2007 crop year were obtained from the University of Arkansas field station at Stuttgart, AR, shipped to the USDA-ARS Center for Grain and Animal Health Research (CGAHR) in Manhattan, KS, and held in cold storage for several months at ∼4°C until used in the experiment. The individual lots of rice were adjusted to 13% moisture content by adding water prior to the initiation of the experiment. The experimental unit consisted of a 7-dram plastic vial (3.5 cm diameter, 6.1 cm height) that could hold 4 g of rough rice. 1 g of rough rice contains approximately 42 kernels; therefore, 4 g of either variety was estimated to be about 160 kernels.

Kernels were examined under a stereomicroscope, and kernels with hulls that were naturally split longitudinally along the transverse axis (hereafter called kernels with cracked hulls) were separated from kernels with intact hulls. The experimental treatments were prepared so that they contained 0, 5, 10, and 20% kernels with cracked hulls in the individual lot of 160 kernels, which was 0, 8, 16, and 32 kernels, respectively. Kernels with cracked hulls were marked by using a pen to make a dot on the exterior of the hull before the introduction of the kernels into the vials. There were four separate replicates for each treatment (combinations of variety and percentage of kernels with cracked hulls).

One- to two-week-old mixed sex adults of *R. dominica* were obtained from laboratory colonies reared on mixed-variety rough rice. Six mixed-sex adults were placed in each vial, and the vials were held for 3 days in an incubator maintained at 32 °C and 70% RH. After this three-day oviposition period, adults were removed, frozen, and sexed by squashing the abdomen to view the genitalia (Potter 1935). The vials were returned to the incubator and held for seven weeks, at which time the vials were removed and the rice sieved to collect F1 progeny adults. The kernels with marked, cracked hulls were examined for adult emergence holes, and the amount of insect frass (indicative of feeding damage) in the vials was also recorded.

Data were analyzed using a two-way analysis of variance (ANOVA), with the percentage of kernels with cracked hulls and the variety as main effects. Response variables were number of F1 progeny, number of kernels with cracked hulls with emergence holes, the proportion of progeny that emerged from kernels with cracked hulls (number of progeny emerged from kernels with cracked hulls/total number of progeny), and the amount of frass. The correlation coefficients were tested in order to determine if the number of parental females in the group of 6 mixed-sex adults affected the number of progeny produced, the number of emergence holes in kernels with cracked hulls, the frass production, or the proportion of progeny emerging from kernels with cracked hulls (Sall et al. 2001). Means were compared using the Tukey-Kramer HSD test at *P* = 0.05 (Sokal and Rohlf 1995).

## Results

For progeny produced, the emergence holes in kernels with cracked hulls, frass production, and proportion of progeny emerging from kernels with cracked hulls variety was significant, but the percentage of kernels with cracked hulls and associated interaction was not (Table 1). The number of females in the group of six parental adults did not affect the number of F1 progeny produced, number of kernels with cracked hulls with emergence holes, the proportion of progeny that emerged from kernels with cracked hulls, or frass production in either variety (respective *r*^2^ values were 0.004, 0.001, 0.01, and 0.002, all *P* values ≥ 0.05, number of females per vial ranged between 2 and 5). Therefore, the number of females was excluded from further analyses. Few progeny were produced on Wells, even in kernels with cracked hulls (Table 2). In samples with 0 and 5% cracked hulls, there were no differences in progeny production, numbers of emergence holes in kernels with cracked hulls, or amount of frass produced in Wells and Francis (Table 2). When the percentage of kernels with cracked hulls increased to 10 and 20%, more progeny, emergence holes, and frass were produced in Francis compared to Wells. However, there was no difference in the three measured parameters between the samples of Francis rice with 10% compared to 20% kernels with cracked hulls. Proportion of progeny emerged from kernels with cracked hulls increased with the percentage of kernels with cracked hulls in the variety Francis, but not Wells. Over 80% of progeny emerged from Francis kernels with cracked hulls when 20% of the kernels had cracked hulls.

## Discussion

The results of our study and those of earlier studies indicate a possible natural tolerance to *R. dominica* development in Wells. In a previous test where only eggs of *R. dominica* were placed in experimental units with Wells rice, few adults emerged (Chanbang et al. 2008a). In another test where parental adult *R. dominica* were placed on different rice varieties from the 2004 crop, including Wells, as part of a study with different commercial diatomaceous earths (Chanbang et al. 2008b), progeny production was poor on untreated Wells rice in comparison with some of the other untreated varieties.

We expected that the number of progeny produced would increase as the percentage of kernels with cracked hulls increased. The lack of increased progeny production in variety Francis as the percentage of kernels with cracked hulls increased from 10 to 20% may be because there was an excess of kernels with cracked hulls at the 20% level relative to the number of eggs laid. At the 10% level where there were 16 kernels with cracked hulls available for oviposition, 50% of progeny emerged from kernels with cracked hulls. At the 20% level where there were 32 kernels with cracked hulls available for oviposition, 84% of progeny emerged from kernels with cracked hulls but only 5 progeny emerged from these kernels; thus, there was a surplus of kernels with cracked hulls. Studies with the rice weevil, *Sitophilus oryzae*, and with the maize weevil, *S. zeamais*, also indicated that infestations were positively correlated with the presence of gaps or splits in the hull (Russell 1968). There were no significant differences in the number of progeny produced in Francis samples with differing proportions of kernels with cracked hulls, and some progeny were produced in samples that contained only kernels with intact hulls. Thus, there is no indication that rice kernels without cracks in the hull are impervious to infestation by *R. dominica*. However, a large proportion of progeny did emerge from kernels with cracked hulls.

Previous studies clearly indicate that presence of kernels with cracked hulls is important in insect reproduction, regardless of the cause of this damage. Breese (1960) found that *R. dominica* adults do not prefer to oviposit in intact kernels, and suggested that the presence of crevices may attract females for egg-laying. Also, varieties with smoother hulls were less susceptible to *R. dominica* than varieties with trichomes or hair-like structures on the hulls (Chanbang et al. 2008b). However, Prakash et al. (1986) noted that the oviposition is not related to hull texture. In our study, the percentage of kernels with cracked hulls was an important variable that affected reproduction by *R. dominica*. Also, the impact of the percentage of kernels with cracked hulls on progeny production was evident at a cracked hull percentage of 10% or higher. Hence, a “critical” proportion of kernels with cracked hulls (here 10%) may play the decisive role in characterizing varietal resistance. In other words, resistant varieties may become susceptible at the existence of this “critical” proportion of kernels with cracked hulls. Conversely, at lower proportions of kernels with cracked hulls a “susceptible” variety may not be susceptible. Therefore, comparison of numerous rice varieties with various proportions of kernels with cracked hulls may not be a reliable estimate to classify varietal resistance. However, this “critical” percentage may not have the same effect among varieties. This “critical” proportion may be different in the case of bulked grain in a commercial warehouse or a bin. In our tests, despite variations in progeny production, the percentage of kernels with cracked hulls did not affect progeny production in Wells, either from kernels with cracked hulls or from intact kernels. Conversely, the percentage of kernels with cracked hulls played an important role in the case of adult emergence from Francis, despite the fact that the increase in progeny production was not proportional to the increase in the number of kernels with cracked hulls.

Other factors besides hull integrity may affect insect reproduction in stored grains. Rice kernel hardness has been correlated with decreased progeny production of other internally-feeding stored-product insects, including *S. oryzae* (Rout et al. 1976). Nutritional composition of rice kernels (Morallo-Rejesus et al. 1982) and differences in kernel composition attributable to field conditions where the rice was grown (Arthur et al. 2009) may affect insect development. In our test, we used a single source for each of the rice varieties; hence, kernel characteristics were not expected to affect progeny production. Thus, it appears that rice kernels with cracked hulls may be more likely to be selected for reproduction by *R. dominica*.

**Table 1.**
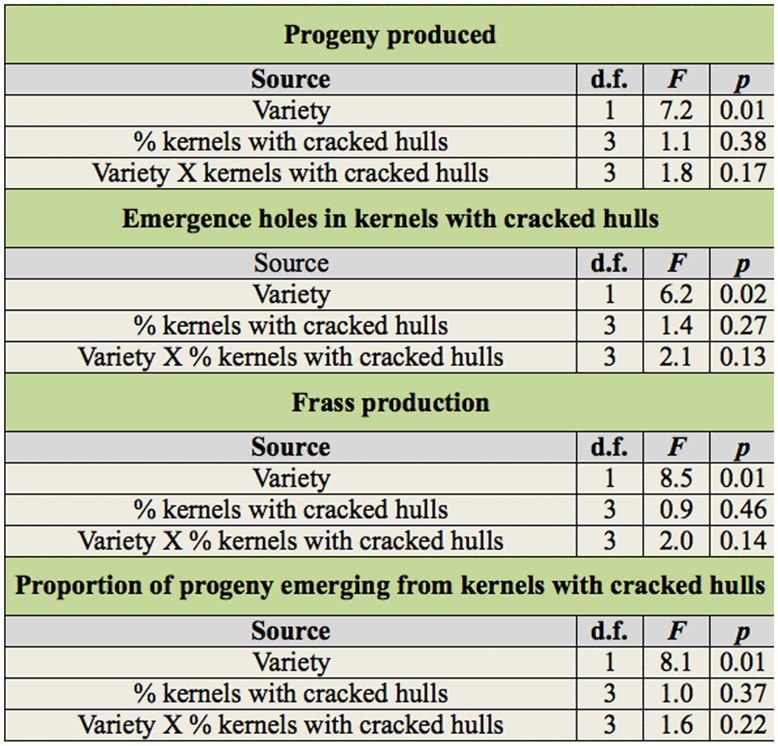
ANOVA parameters for main effects and associated interactions for progeny produced, emergence holes in kernels with cracked hulls, frass production, and proportion of progeny emerging from kernels with cracked hulls of *Rhyzopertha* *dominica* adults (in all cases total d.f. = 31).

**Table 2.**
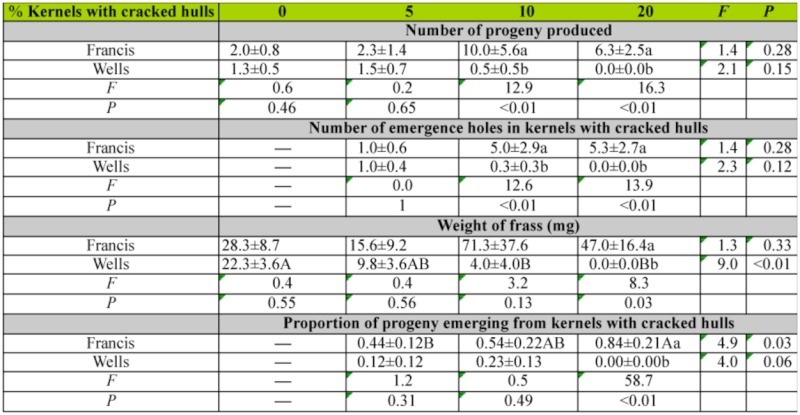
Mean ± SE number of *Rhyzopertha dominica* progeny, number of emergence holes in rough rice kernels of the varieties Francis and Wells with cracked hulls, weight of frass, and proportion of progeny emerging from kernels with cracked hulls in 4 g of rough rice (∼ 160 kernels) containing 0, 5, 10, 15, and 20% kernels with cracked hulls. Assessments were made 49 days after the removal of six mixed-sex parental *R. dominica* adults. Means within columns followed by the same lower case letter, d.f. = 1, 7, or within rows followed by the same uppercase letter, d.f. = 3, 15, are not significantly different. Where no letters exist no significant differences were noted.

## References

[bibr01] Amos TG, Semple RL, Williams P (1986). Multiplication of some stored grain insects on varieties of wheat.. *General and Applied Entomology*.

[bibr02] Arthur FH, Bautista RC, Siebenmorgen TJ (2009). Influence of growing location and cultivar on *Rhyzopertha dominica* (Coleoptera: Bostrichidae) and *Sitophilus oryzae* (Coleoptera: Curculionidae) infestation of rough rice.. *Insect Science*.

[bibr03] Athanassiou CG, Opit GP, Throne JE (2010). Influence of commodity type, percentage of cracked kernels, and wheat class on population growth of stored-product psocids (Psocoptera: Liposcelidae).. *Journal of Economic Entomology*.

[bibr04] Bhatia SK, Gupta M (1969). Resistance to stored grain pests in world collection of wheat-relative susceptibility of nine high yielding dwarf varieties to the rice weevil and the lesser grain borer.. *Bulletin of Grain Technology*.

[bibr05] Breese MH (1960). The infestibility of stored paddy by *Sitophilus sasakii* (Tak.) and *Rhizopertha dominica* (F.).. *Bulletin of Entomological Research*.

[bibr06] Chanbang Y, Arthur FH, Wilde GE, Throne JE (2008a). Control of *Rhyzopertha dominica* in stored rough rice through a combination of diatomaceous earth and varietal resistance.. *Insect Science*.

[bibr07] Chanbang Y, Arthur FH, Wilde GE, Throne JE (2008b). Hull characteristics as related to susceptibility of different varieties of rough rice to *Rhyzopertha dominica* (F.) (Coleoptera: Bostrichidae).. *Journal of Stored Products Research*.

[bibr08] Cogburn RR (1974). Domestic rice varieties: apparent resistance to rice weevils, lesser grain borers and Angoumois grain moths.. *Environmental Entomology*.

[bibr09] McGaughey WH, Speirs RD, Martin CR (1990). Susceptibility of classes of wheat grown in the United States to stored-grain insects.. *Journal of Economic Entomology*.

[bibr10] Morallo-Rejesus B, Javier PA, Juliano BO (1982). Properties of brown rice and varietal resistance to storage insects.. *Phillipine Entomologist*.

[bibr11] Pomeranz Y, Czuchajowska Z, Shogren MD, Rubenthaler GL, Bolte LC, Jeffers HC, Mattern PJ (1988). Hardness and functional (bread and cookie-making) properties of U.S. wheats.. *Cereal Foods World*.

[bibr12] Potter C (1935). The biology and distribution of *Rhyzopertha dominica* (Fab.).. *Transactions of the Royal Entomological Society of London*.

[bibr13] Prakash A, Pasalu IC, Mathur KC (1986). Oviposition and development of lesser grain borer and rice weevil in relation to some morphological rice grain characters.. *Indian Journal of Agricultural Research*.

[bibr14] Rout G, Senapati B, Ahmed T (1976). Studies on relative susceptibility of some high yielding varieties of rice to the rice weevil, *Sitophilus oryzae* L. (Curculionidae: Coleoptera).. *Bulletin of Grain Technology*.

[bibr15] Russell MP (1968). Influence of rice variety on oviposition and development of the rice weevil, *Sitophilus oryzae*, and the maize weevil, *S. zeamais*.. *Annals of the Entomological Society of America*.

[bibr16] Sall J, Lehman A, Creighton L (2001). *JMP Start Statistics. A guide to statistics and data analysis using JMP and JMP IN software*..

[bibr17] Sinha RN, Demianyk CJ, McKenzie RIH (1988). Vulnerability of common wheat cultivars to major stored-product beetles.. *Canadian Journal of Plant Science*.

[bibr18] Sokal RR, Rohlf FJ (1995). *Biometry*.

[bibr19] Throne JE, Baker JE, Messina FJ, Kramer KJ, Howard JA, Subramanyam B, Hagstrum DW (2000). Varietal resistance.. *Alternatives to Pesticides in Stored product IPM*..

[bibr20] Towes MD, Cuperus GW, Phillips TW (2000). Susceptibility of eight U.S. wheat cultivars to infestation by *Rhyzopertha dominica* (Coleoptera: Bostrichidae).. *Environmental Entomology*.

